# Identification of an Amphipathic Helix Important for the Formation of Ectopic Septin Spirals and Axial Budding in Yeast Axial Landmark Protein Bud3p

**DOI:** 10.1371/journal.pone.0016744

**Published:** 2011-03-08

**Authors:** Jia Guo, Ting Gong, Xiang-Dong Gao

**Affiliations:** State Key Laboratory of Virology, College of Life Sciences, Wuhan University, Wuhan, China; Dartmouth College, United States of America

## Abstract

Correct positioning of polarity axis in response to internal or external cues is central to cellular morphogenesis and cell fate determination. In the budding yeast *Saccharomyces cerevisiae*, Bud3p plays a key role in the axial bud-site selection (axial budding) process in which cells assemble the new bud next to the preceding cell division site. Bud3p is thought to act as a component of a spatial landmark. However, it is not clear how Bud3p interacts with other components of the landmark, such as the septins, to control axial budding. Here, we report that overexpression of Bud3p causes the formation of small septin rings (∼1 µm in diameter) and arcs aside from previously reported spiral-like septin structures. Bud3p closely associates with the septins *in vivo* as Bud3p colocalizes with these aberrant septin structures and forms a complex with two septins, Cdc10p and Cdc11p. The interaction of Bud3p with the septins may involve multiple regions of Bud3p including 1–858, 850–1220, and 1221–1636 a.a. since they all target to the bud neck but exhibit different effects on septin organization when overexpressed. In addition, our study reveals that the axial budding function of Bud3p is mediated by the N-terminal region 1–858. This region shares an amphipathic helix (850–858) crucial for bud neck targeting with the middle portion 850–1103 involved in the formation of ectopic septin spirals and rings. Interestingly, the Dbl-homology domain located in 1–858 is dispensable for axial bud-site selection. Our findings suggest that multiple regions of Bud3p ensure efficient targeting of Bud3p to the bud neck in the assembly of the axial landmark and distinct domains of Bud3p are involved in axial bud-site selection and other cellular processes.

## Introduction

The establishment of a proper polarity axis in response to internal or external cues is essential for morphogenesis, cell fate determination, and directional cell migration in many types of cells [1], [2]. In the budding yeast *Saccharomyces cerevisiae*, determination of the new polarity axis, a process also known as bud-site selection, occurs prior to the assembly of a new bud and how cells choose the new budding site varies among cell types [3]. Haploid *MAT*a or *MAT*α cells choose the new site next to the preceding cell division site. This pattern is called axial. Diploid *MAT*a/*MAT*α cells, however, position the new site distal to the preceding division site. This pattern is called bipolar. Genetic studies revealed that two sets of gene products are implicated in the determination of the axial pattern (Axl1p, Axl2p, Bud3p, Bud4p, and the septins) [4], [5], [6], [7], [8], [9] and the bipolar pattern (Bud8p, Bud9p, Rax1p, and Rax2p) [10], [11], [12], [13]. These two sets of proteins can activate the Ras-family GTPase Rsr1p by interacting with the Rsr1p GTPase module consisting of Rsr1p and its regulators Bud2p (GTPase-activating protein) and Bud5p (guanine nucleotide exchange factor), which in turn activates the Rho-family GTPase Cdc42p, leading to the assembly of a new bud at a chosen site.

Bud3p plays a key role in axial budding as *bud3* mutants do not bud in an axial pattern but rather bud in a bipolar pattern [6]. It is thought that Bud3p and three other axial budding determinants Axl1p, Axl2p, and Bud4p anchor to the septin filaments at the bud neck and together they form the axial landmark [6], [9], [14], [15]. Bud3p expression is cell cycle regulated. It peaks near the onset of mitosis [16]. Bud3p shows a localization pattern similar to that of the septins and other axial budding determinants: It first localizes to the bud neck as a collar. At the time of cytokinesis, the collar splits into two single rings sandwiching the bud neck. After cell separation, the daughter cell and the mother cell each inherit one ring, which persists for a while until the new bud starts to form [6], [17]. This ring with the axial budding determinants on it serves as a spatial landmark for the cell to position the new budding site in the next cell cycle. Several studies suggest that the axial landmark is assembled in a sequential order. The bud neck localized septin filaments act as the core component of this landmark [4]. Bud3p and Bud4p are recruited to the bud neck by the septin filaments [6], [9], which further recruit the other two components Axl1p and Axl2p to the bud neck [7], [14], [15]. However, the detailed interactions between the components of the axial landmark are not well understood.

Bud3p is expressed in both haploid and diploid yeast cells. However, the latter do not bud in the axial pattern. This implies that Bud3p may have a general function rather than merely controls axial budding. *bud3*Δ strains in certain genetic background displayed an increase in the population of cells with two buds on one mother cell [8], [18], suggesting that Bud3p may play a role in cytokinesis. Compared to Bud3p, Bud3p orthologs in *Ashbya gossypii* (a filamentous yeast) and two filamentous ascomycete fungi *Neurospora crassa* and *Aspergillus nidulans* also localize to the incipient septation sites as a ring but play a more prominent role than Bud3p in septum formation [19], [20], [21]. A previous study showed that high-levels of Bud3p exhibited deleterious effect on cell morphology and septin organization [16]. Cells displayed large and long buds and were connected in chains. Septins at the bud neck were often absent or poorly organized. In the elongated buds, septins mislocalized to a patch at the bud tips or to spiral-like multiple rings (referred hereafter as spirals) on the periphery of buds extending from the bud neck to bud tip. This finding suggests that Bud3p may play a role in septin organization and supports a role for Bud3p in cytokinesis.

Bud3p lacks recognizable domains that can help infer its function except the region 259–442 at the N-terminus, which shows weak homology to Dbl-homology (DH) domain commonly found in guanine nucleotide exchange factors (GEF) for the Rho GTPases [22]. It is not clear if Bud3p functions as a Rho GEF and, if so, which Rho GTPase is the substrate. Bud3p orthologs can be identified in most yeast species and filamentous fungi and they all contain the signature DH domain [19], [21]. The homologous region in Bud3p orthologs locates in the N-terminal region of the protein comprising the DH domain whereas the C-terminal regions show great variation. The presence of a DH domain in Bud3p and its orthologs suggests that this domain may be important for their function. Recent studies on Bud3p orthologs in *N. crassa* and *A. nidulans* revealed that the region comprising the DH domain exhibits guanine nucleotide exchange factor (GEF) activity for the Rho GTPase Rho4 *in vitro* and this activity is critical for the assembly of the actomyosin ring at the cytokinetic sites [20], [21].

In this study, we show that Bud3p is closely associated with the sepins as it colocalizes with aberrant septin structures upon overexpression and forms a complex *in vivo* with two septins. While multiple regions of Bud3p could target to the bud neck, only the N-terminal portion of Bud3p is capable of carrying out the axial budding function. Moreover, we identify an amphipathic helix important for axial budding and the formation of ectopic septin spirals, and the DH domain is dispensable for Bud3p's function in axial budding. Our findings provide new insights in understanding the role of Bud3p in the assembly of the axial landmark.

## Results

### Bud3p localizes to ectopic septin spirals and rings upon overexpression

**Figure 1:**
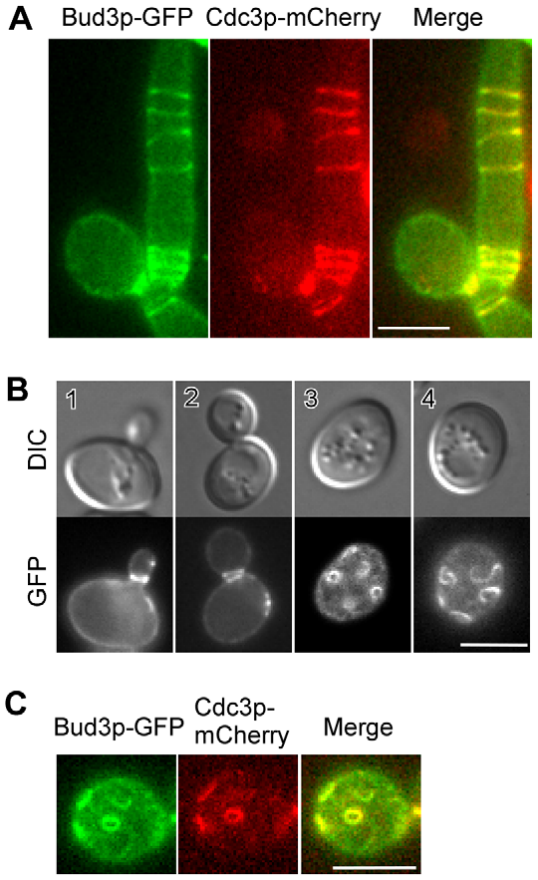
Bud3p localizes to ectopic septin spirals and rings upon overexpression. (**A**) Bud3p colocalizes with the septin spirals. Cells of strain JGY1783 (a *CDC3-mCherry*) carrying plasmid pUG36-BUD3 (expresses Bud3p-GFP) were grown on SC-Ura medium and imaged by two-color fluorescence microscopy. (**B**) Bud3p localizes to the plasma membrane and ectopic rings, short bars, and arcs. Cells of strain YEF3570 (a *bud3*Δ) carrying plasmid pUG36-BUD3 were imaged. Cells 1 and 2 were focused on the equatorial plane whereas cells 3 and 4 were focused near the top surface. (**C**) The ectopic Bud3p rings contain the septins. Cells as shown in A were imaged by two-color fluorescence microscopy. Bar, 5 µm.

Previous studies have shown that Bud3p and the septin cytoskeleton interact genetically. Particularly, overexpression of Bud3p causes extensive bud elongation and the formation of septin spirals in the elongated buds [16]. To further explore the relationship of Bud3p with the septins, we asked whether Bud3p is present in those septin spirals. To this end, we took advantage of a plasmid-borne *BUD3-GFP* construct that expresses Bud3p with a GFP tag at its N-terminus under the control of *MET25* promoter. This construct is functional as it complemented the axial budding defect of haploid *bud3*Δ cells (Table 1, BUD3). When it was introduced into yeast cells that also express Cdc3p-mCherry (a septin tagged with a red fluorescent protein), a fraction (∼10%) of cells displayed elongated buds due to overexpression of Bud3p. In those elongated buds that displayed Cdc3p-mCherry spirals on the bud cortex, Bud3p colocalized with Cdc3p in these structures (Fig. 1A).

**Table 1:** Budding pattern of strain YEF3570 (a *bud3*Δ) expressing pUG36-BUD3 fragments.

BUD3 constructs	Axial(%)	Bipolar (%)	Random (%)
Vector	19	61	20
BUD3	83	9	8
BUD3-FLΔDH	66	17	17
BUD3-NM	58	24	18
BUD3-MC	23	62	15
BUD3-N	24	58	18
BUD3-M	21	56	23
BUD3-C	21	55	24

In those ∼90% cells with a normal morphology, Bud3p-GFP localized to the bud neck (Fig. 1B, cells 1–2), in agreement with previous observations [6], [17]. Bud3p was also found on the plasma membrane (shortened as PM hereafter) (Fig. 1B, cells 1–2), a localization not reported before. This localization may result from protein overexpression because we failed to detect similar localization in yeast strains with their endogenous *BUD3* gene tagged by GFP (data not shown). Surprisingly, we also observed Bud3p-GFP in cortical structures resembling small rings and short arcs (Fig. 1B, cells 3–4). These structures are located on the cell surface and are often observed in cells with a normal morphology. They can also be detected in cells with elongated buds but they tend to locate in the mother cells rather than in the elongated buds. These structures appear to be randomly distributed and their numbers in each cell varied. The small rings are fairly uniform in size with a diameter of ∼1 µm. Remarkably, they also contained the septins as Bud3p-GFP and Cdc3p-mCherry displayed a colocalization in these structures (Fig. 1C). The ability of excess Bud3p to cause the formation of ectopic septin spirals and small rings and the association of Bud3p with these abnormal septin structures suggest that Bud3p and the septins are closely associated.

### The middle portion of Bud3p is involved in the formation of ectopic septin spirals

**Figure 2:**
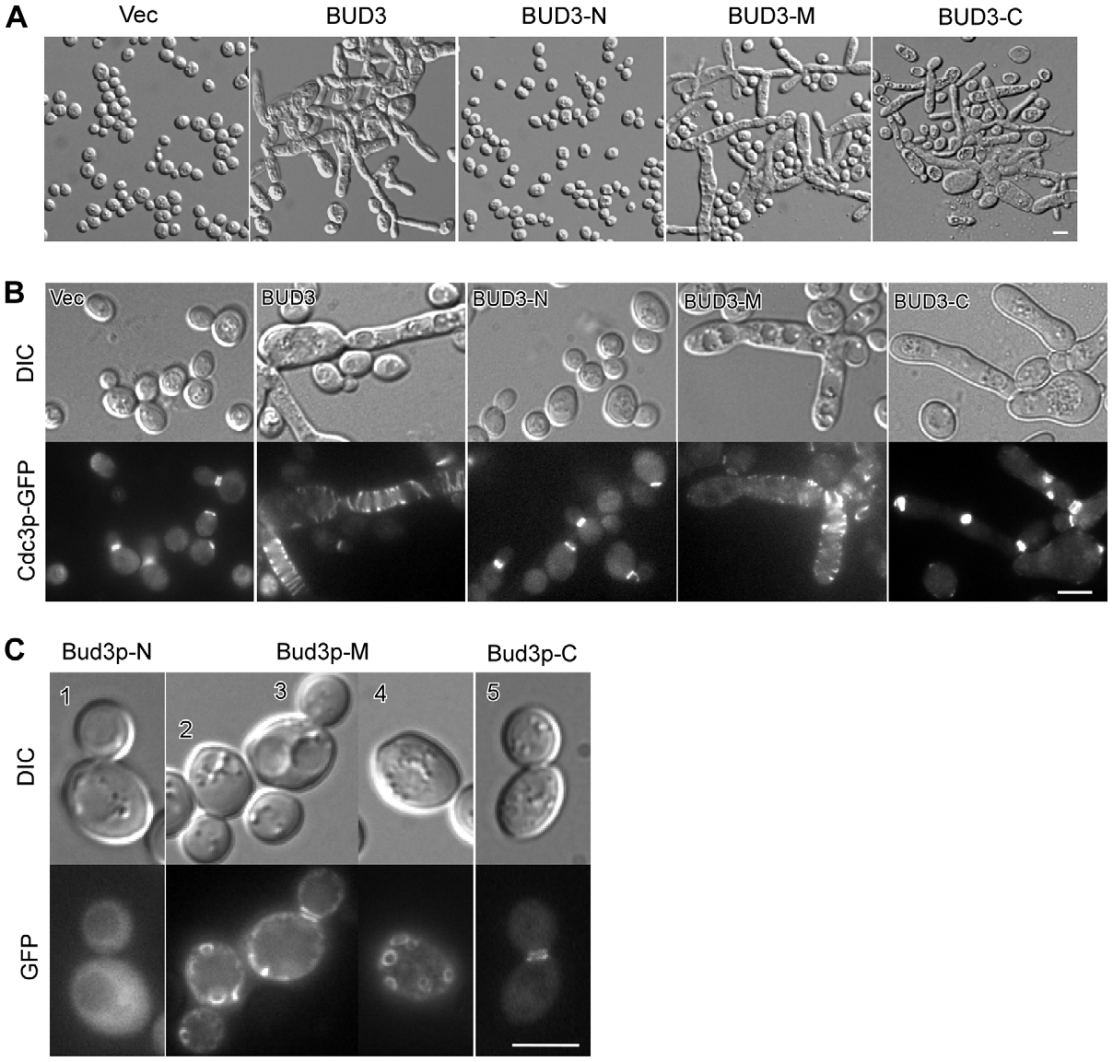
The middle portion of Bud3p is involved in the formation of ectopic septin spirals. (**A**, **B**) Overexpression of Bud3p-M or Bud3p-C caused defects in cell morphology (**A**) and septin organization (**B**). Strain JGY881 (a *CDC3-GFP*) carrying plasmids pEGKT316 (vector), pEGKT316-BUD3, or pEGKT316-BUD3 fragments was grown on SC-Ura medium containing 2% galactose and 1% raffinose at 30°C. Images were taken after 2 d. Cdc3p-GFP localization was examined (**B**). (**C**) Subcellular localization of Bud3p fragments. Cells of strain YEF3570 (a *bud3*Δ) carrying plasmid pUG36-BUD3 fragments were imaged. Note cell #4 was focused near the top surface. Bar, 5 µm.

Bud3p is a large protein of 1636 amino acids. To explore how Bud3p overexpression leads to bud elongation, and particularly, how ectopic septin spirals are formed, we set out to determine the functional region of Bud3p responsible for the formation of these structures. To this end, we divided Bud3p into three pieces: Bud3p-N (1–673), Bud3p-M (674–1220), and Bud3p-C (1221–1636), and examined each of them for their ability to cause bud elongation and induce the formation of ectopic septin structures. These fragments were tagged with GST at their N-terminus and expressed under the control of a galactose-inducible promoter. Overexpression of Bud3p-N did not affect cell morphology or septin organization (Fig. 2A, 2B). In contrast, overexpression of either Bud3p-M or Bud3p-C greatly altered cell morphology (Fig. 2A). In both cases, many cells displayed elongated buds and cell chains, similar to overexpression of full-length Bud3p. Septin organization in these cells was also defective as shown by Cdc3p-GFP (Fig. 2B). However, ectopic septin spirals were only detected in cells overexpressing Bud3p-M but not in cells overexpressing Bud3p-C. The septins in the latter were often mislocalized to a patch at the tip or on the side of elongated buds (Fig. 2B). These results suggest that two distinct regions of Bud3p, the middle portion (674–1220) and the C-terminal portion (1221–1636), both can interact with the septins, either directly or indirectly. But upon overproduction, only the middle portion of Bud3p is able to cause the formation of ectopic septin spirals.

Next, we investigated the subcellular localization of these Bud3p fragments by expressing them as GFP-fusion proteins in *bud3*Δ cells under the control of *MET25* promoter. We found that, among these fragments, only Bud3p-M localized to small rings, short arcs, and bars on the cell surface (Fig. 2C, cells 2–4). The other two fragments either stayed in the cytoplasm (Bud3p-N) or localized to the bud neck as a double ring in large-budded cells (Bud3p-C) (Fig. 2C). Bud3p-M also localized to the bud neck in large-budded cells and displayed a PM localization similar to full-length Bud3p (Fig. 2C, cell 3). Immunoblotting with anti-GFP antibodies showed that these Bud3p-GFP fragments were properly expressed (Fig. S1).

Together, our results indicate that, among the three Bud3p fragments, only Bud3p-M resembles full-length Bud3p in terms of their ability to induce bud elongation, the formation of ectopic septin spirals and rings, and PM localization.

### Septin spiral formation is mediated by the region 850–1103 comprising an amphipathic helix

**Figure 3:**
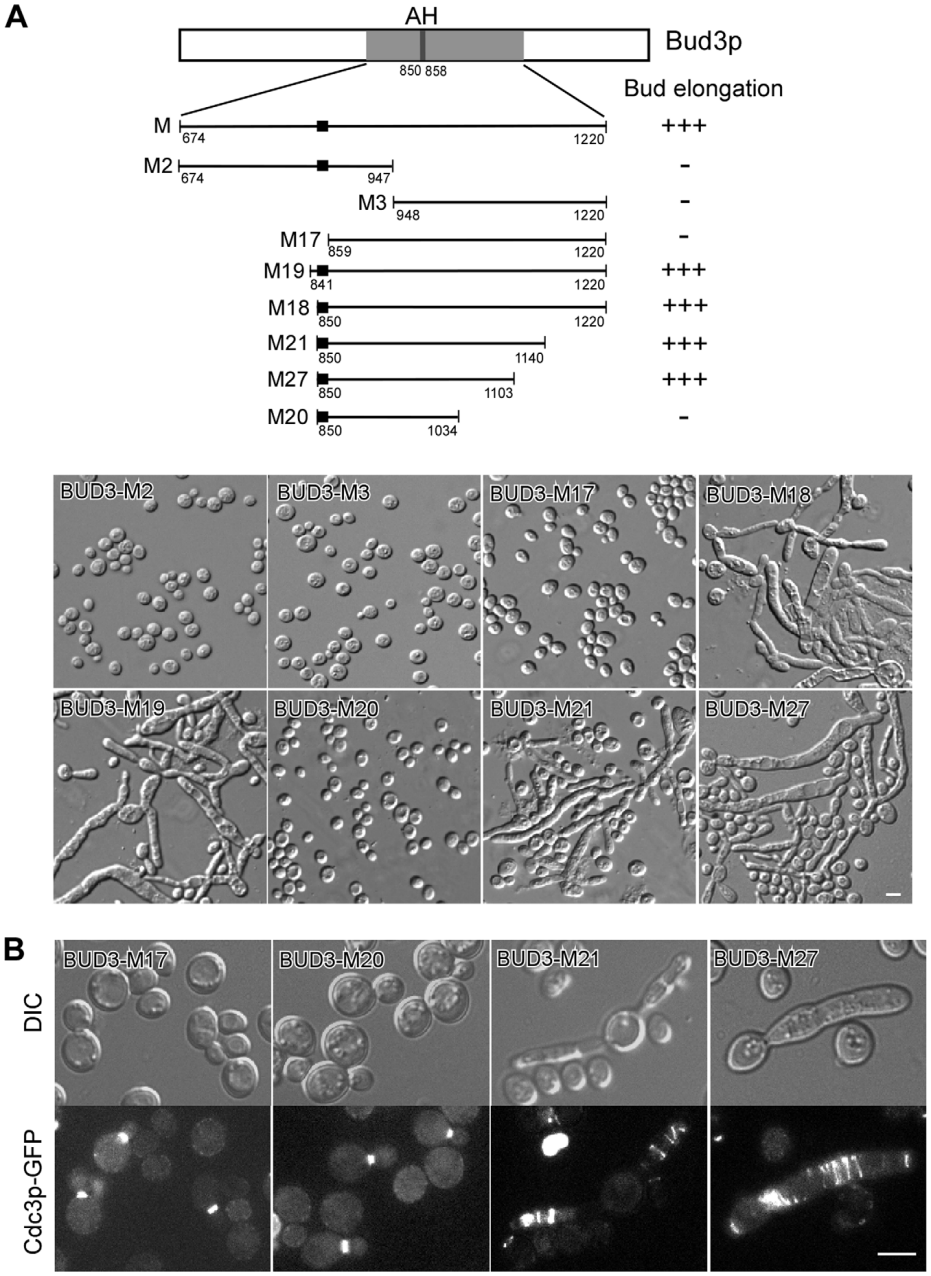
Characterization of the shortest functional region responsible for septin spiral formation. (**A**) The morphology of cells overexpressing truncated Bud3p-M fragments. Strain JGY881 (a *CDC3-GFP*) carrying plasmids pEGKT316-BUD3 fragments was grown on SC-Ura medium containing 2% galactose and 1% raffinose at 30°C. DIC images were taken after 2 d. Upper panel, defect in bud morphology. Lower panel, representative cell morphology. (**B**) Representative cells with Cdc3p-GFP localization in (**A**) were shown. Bar, 5 µm.

To delineate the functional region in Bud3p-M responsible for bud elongation and septin spiral formation, we constructed a series of truncated Bud3p-M and overexpressed them. Among the N-terminal truncated fragments, two longer ones, Bud3p-M19 (841–1220) and Bud3p-M18 (850–1220), were able to cause bud elongation and the formation of cell chains, while the three shorter ones, Bud3p-M2 (674–947), Bud3p-M3 (948–1220), and Bud3p-M17 (859–1220), were not (Fig. 3A). Thus, the N-terminal boundary of the functional domain was placed at residue 850.

Among the C-terminal truncated fragments, the two longer ones, Bud3p-M21 (850–1140) and Bud3p-M27 (850–1103), caused bud elongation and the formation of cell chains (Fig. 3A). In the elongated buds, ectopic septin spirals were observed as shown by Cdc3p-GFP (Fig. 3B), suggesting that both of them are fully functional. In contrast, the shorter fragment Bud3p-M20 (850–1034) failed to cause bud elongation (Fig. 3A) or septin spiral formation (Fig. 3B). Thus, we have identified the region 850–1103 in Bud3p-M as the functional region capable of causing bud elongation and septin spiral formation upon overexpression.

**Figure 4:**
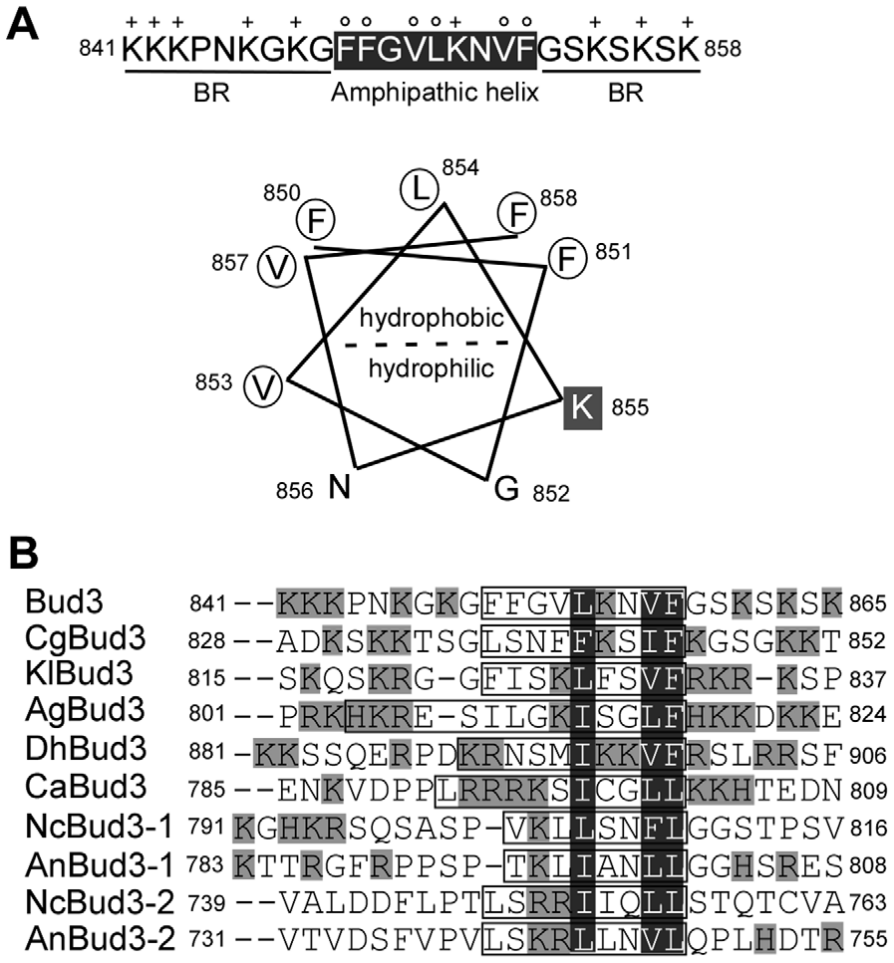
Bud3p contains a sequence that potentially forms an amphipathic helix. (**A**) Upper panel, the amino acid sequence of the amphipathic helix (850–858) and flanking basic-rich (BR) motifs. Positively charged and hydrophobic residues are indicated with + and o, respectively. Lower panel, a helical wheel projection of the amphipathic helix. (**B**) Sequence alignment of amphipathic helices and flanking BR motifs found in Bud3p orthologs from yeasts and filamentous fungi. Cg, *Candida glabrata*; Kl, *Kluyveromyces lactis*; Ag, *Ashbya gossypii*; Dh, *Debaryomyces hansenii*; Ca, *Candida albicans*; Nc, *Neurospora crassa*; An, *Aspergillus nidulans*. The sequences marked in boxes are the predicted amphipathic helices. The positively charged and hydrophobic residues are marked in grey and black shade, respectively. NcBud3 and AnBud3 have two amphipathic helices.

The region 850–1103 lacks recognizable domains that can provide clues to its function. Sequence alignment with Bud3p orthologs found in other yeast species also failed to identify highly homologous sequence in this region. However, there is a short sequence, ^850^FFGVLKNVF^858^, at its N-terminus that resembles an amphipathic helix (Fig. 4A) [23], [24]. This sequence is predicted to form an α-helix by the Swiss-Model Workspace (http://swissmodel.expasy.org/workspace/). When projected on a helical wheel, one half of the helix is highly hydrophobic and consists of phenylalanine, valine, and leucine residues, whereas the other half is hydrophilic and contains one positively charged lysine residue (Fig. 4A, lower panel). In addition, multiple positively charged residues, mostly lysines, are present in the sequences flanking the helix. The helix and the positively charged residues in the flanking sequences appear to be evolutionarily conserved in Bud3p orthologs found in yeast species including *Kluyveromyces lactis*, *Candida albicans*, and *Debaryomyces hansenii* as well as two filamentous ascomycete fungi *Neurospora crassa* and *Aspergillus nidulans* (Fig. 4B). We hereafter refer to this helix as amphipathic helix and the flanking sequences rich in basic residues as basic-rich (BR) motifs.

### The amphipathic helix 850–858 and adjacent BR motifs confer PM targeting to Bud3p and is critical for septin spiral formation

**Figure 5:**
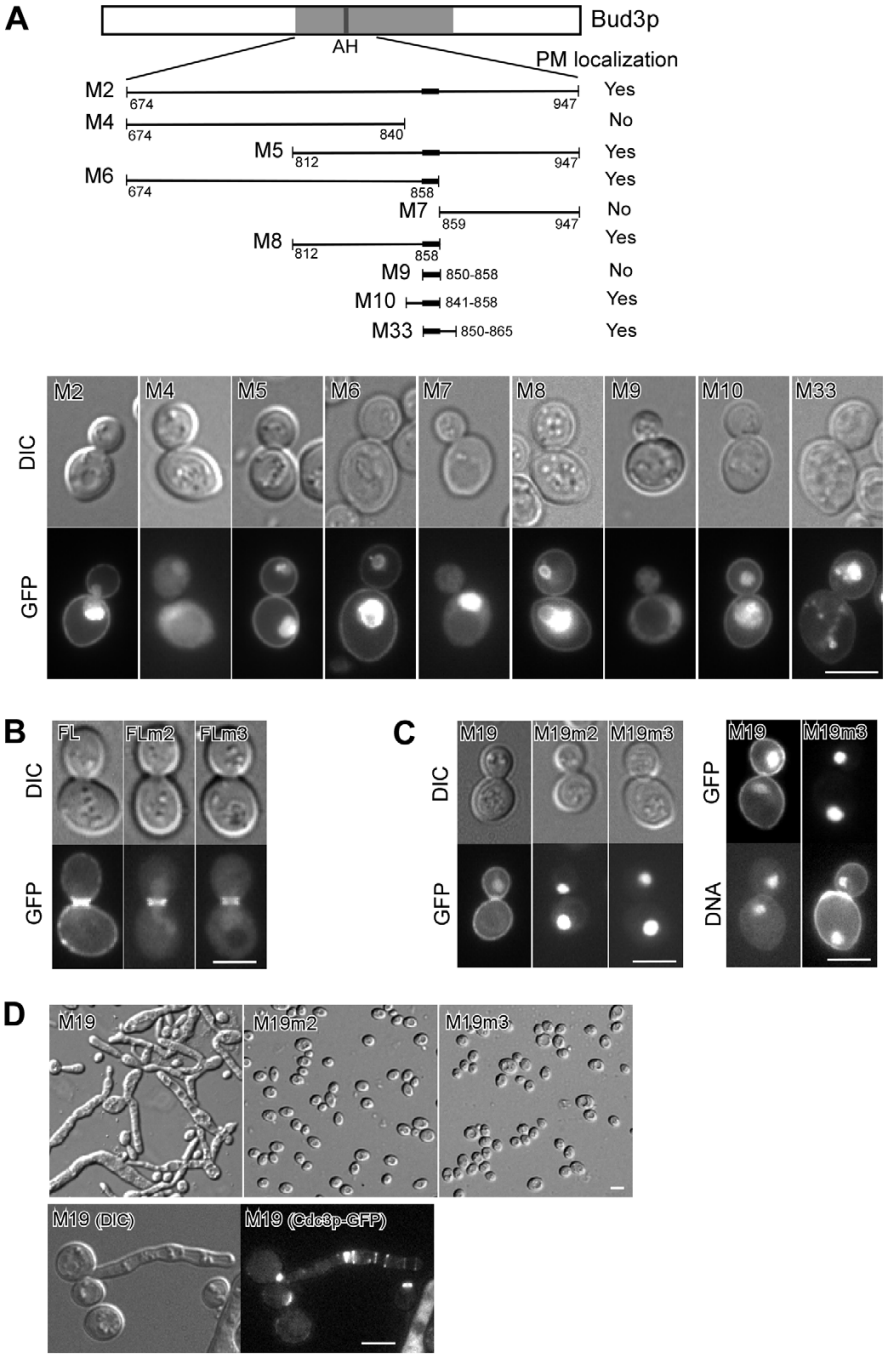
The amphipathic helix confers PM targeting to Bud3p and is critical for septin spiral formation. (**A**) Bud3p fragments carrying the amphipathic helix and flanking BR motifs could localize to the PM. Cells of strain YEF3570 (a *bud3*Δ) carrying plasmid pUG36-BUD3 fragments that express GFP-fusion proteins were imaged. Upper panel, Bud3p fragments and their localization. Lower panel, representative localization. (**B**) Mutations of key residues in the amphipathic helix abolished PM targeting of full-length Bud3p. Cells of strain YEF3570 (a *bud3*Δ) carrying plasmid pUG36-BUD3-FL (full-length) or mutants (-FLm2 and -FLm3) were examined for localization of GFP-fusion proteins. Mutated sequences are m2 (850-aaGaaKNVF-858) and m3 (850-FFGVLKNaa-858). “a” stands for alanine. (**C, D**) Mutations of key residues in the amphipathic helix abolished PM targeting of Bud3p-M19 (841-1220) (**C**), bud elongation (**D**, top), and septin spiral formation (**D**, bottom). In (**C**) right panel, cells were stained for DNA with Hoechst dye. In (**D**), strain JGY881 (a *CDC3-GFP*) carrying plasmid pEGKT316-BUD3-M19 or BUD3-M19 mutants was grown on SC-Ura medium containing 2% galactose and 1% raffinose. Bar, 5 µm.

It is known that amphipathic helices confer PM targeting to some proteins [24], [25], [26]. The fact that a pool of Bud3p localized to the PM raised a possibility that Bud3p's PM localization could be mediated by the amphipathic helix 850–858. Indeed, we found that truncated versions of Bud3p-M that carry this helix, such as Bud3p-M2 (674–947), Bud3p-M6 (674–858), and Bud3p-M8 (812–858), localized to the PM, whereas fragments lacking this helix, such as Bud3p-M4 (674–840) and Bud3p-M7 (859–947), did not exhibit PM localization (Fig. 5A). Furthermore, replacement of the hydrophobic residues in the amphipathic helix with alanines in full-length Bud3p abolished PM targeting as Bud3p-FLm2 (F850-851A, V853A, L854A) and Bud3p-FLm3 (V857A, F858A) mutants completely failed to localize to the PM (Fig. 5B). These results indicate that the amphipathic helix 850–858 is crucial for targeting Bud3p to the PM.

Surprisingly, we found that the amphipathic helix alone (850–858) did not target to the PM (Fig. 5A, Bud3p-M9). However, the amphipathic helix plus either the left BR motif ^841^KKKPNKGKG^849^ (Bud3p-M10, 841–858) or the right BR motif ^859^GSKSKSK^865^ (Bud3p-M33, 850–865) together could localize to the PM (Fig. 5A). The BR motifs are rich in positively charged lysines. This finding suggests that two BR motifs may act together to help the amphipathic helix anchor on the PM by interacting electrostatically with the anionic phospholipids at the cytoplasmic leaflet of the PM.

Bud3p-M19 (841–1220), a fragment of Bud3p-M that carries the amphipathic helix, localized to the PM and the bud neck (Fig. 5C) and caused bud elongation and septin spiral formation upon overexpression (Fig. 5D). However, Bud3p-M19m2 (F850-851A, V853A, L854A) and Bud3p-M19m3 (V857A, F858A), two Bud3p-M19 mutants that carry alanine substitutions for the hydrophobic residues in the amphipathic helix, both failed to localize to the PM (Fig. 5C) and did not cause bud elongation when overexpressed (Fig. 5D, upper row), demonstrating that the amphipathic helix 850–858 is critical for septin spiral formation. These mutants also lost their localization to the bud neck (Fig. 5C), implying that the formation of ectopic septin spirals by excess Bud3p-M19 depends on its ability to interact with the bud neck-localized septins. Accordingly, the amphipathic helix is likely involved in the interaction between Bud3p and the septins.

Interestingly, Bud3p-M19m2 and Bud3p-M19m3 mutants showed prominent nuclear localization (Fig. 5C, right panel), suggesting that mutations that diminished PM binding strengthened nuclear targeting.

### Bud3p interacts with the septins *in vivo*

**Figure 6:**
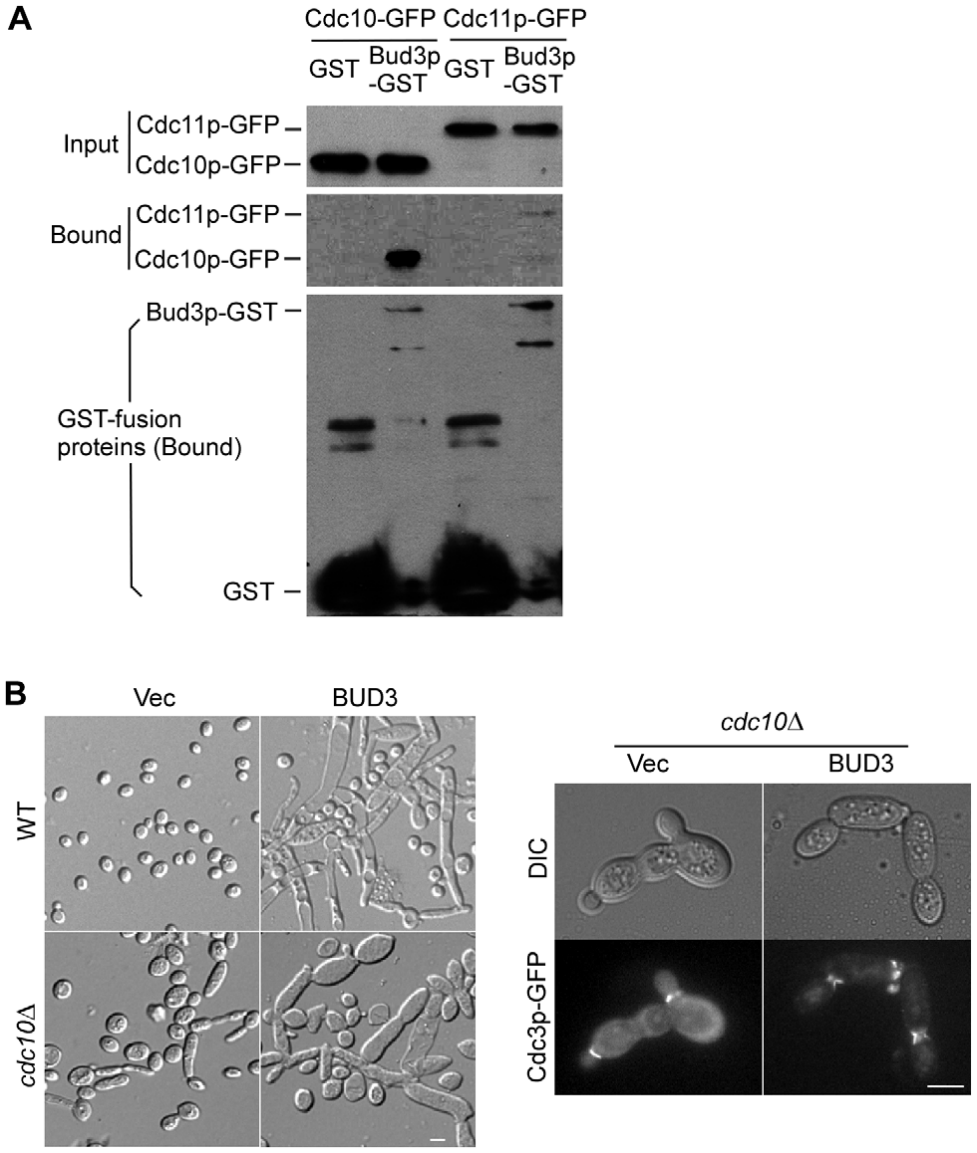
Bud3p interacts with the septins *in vivo*. (**A**) GST pull-down assay. Strains of JGY2019 (a *bud3*Δ *CDC10-GFP*) and JGY2081 (a *bud3*Δ *CDC11-GFP*) carrying plasmid pEGKT316 (GST) or pEGKT316-BUD3 (Bud3p-GST) were used. Cdc10p-GFP or Cdc11p-GFP in the cell lysates (Input) and in the pull-down precipitates (Bound) were analyzed by SDS-PAGE and immunoblotted with an anti-GFP antibody. GST-tagged proteins in the pull-down precipitates (Bound) were immunoblotted with an anti-GST antibody. (**B**) Cdc10p is necessary for septin spiral formation but not for bud elongation. Left panel: Yeast strains YEF473A (WT, wild-type) and YEF4601 (*cdc10*Δ) carrying empty pEGKT316 vector (Vec) or pEGKT316-BUD3 (BUD3) were grown on SC-Ura plates containing 2% galactose and 1% raffinose at 24°C. Right panel: Strain YEF4601 (*cdc10*Δ) carrying YIplac128-CDC3-GFP was transformed with pEGKT316 vector (Vec) and pEGKT316-BUD3 (BUD3). Cdc3p-GFP localization was visualized in transformants grown on SC-Ura plates containing galactose and raffinose at 24°C. Bars, 5 µm.

The colocalization of Bud3p with the septins in abnormal septin spirals and rings raises the possibility that Bud3p may interact with one or more septins *in vivo*. We employed GST pull-down assay to assess this possibility. All the five mitotic septins were tagged with GFP in haploid *bud3*Δ cells. Bud3p was tagged with GST and expressed on a low-copy plasmid under the control of *GAL1_UAS_*-*CYC1_P_* hybrid promoter. Bud3p-GST was pulled down using glutathione beads and the presence of GFP-tagged septins was probed by immunoblotting with anti-GFP antibodies. GST pull-down experiments showed that Bud3p coprecipitated with two septins, Cdc10p and Cdc11p, particularly more effective with Cdc10p (Fig. 6A). We failed to detect coprecipitation of Bud3p with other three septins, Cdc3p, Cdc12p, and Shs1p (data not shown).

Cdc10p plays an important role in the organization of higher-order septin structures. Deletion of *CDC10* is known to affect the localization of other septins at the bud neck [27]. We found that Bud3p overexpression in *cdc10*Δ cells still caused bud elongation and cell chain formation (Fig. 6B, left panel), but the effect was less robust than in wild-type control. In those cells that displayed the septins at the bud neck as shown by Cdc3p-GFP, the septins often appeared as patches or vertical bars at the bud neck, more defective than in control cells (Fig. 6B, right panel). This implies that Bud3p might have additional interacting partners other than Cdc10p at the bud neck. Interestingly, we found that no ectopic septin spirals could be detected in *cdc10*Δ cells overexpressing Bud3p (Fig. 6B, right panel), indicating that Cdc10p is necessary for the formation of septin spirals.

### Bud3p's function in axial budding relies on the N-terminal region that slightly overlaps with the middle portion involved in septin spiral formation

Bud3p is a key player in axial bud-site selection. We wanted to identify the functional region in Bud3p responsible for controlling axial budding. As the plasmid-borne GFP-fusion construct of full-length Bud3p efficiently restored haploid *bud3*Δ cells from a defective bipolar budding pattern to a normal axial budding pattern (Table 1), we set out to study whether some of the Bud3p truncation mutants could rescue the axial budding defect of *bud3*Δ cells. We found that Bud3p-NM (1-1220) could restore axial budding to *bud3*Δ cells, but none of Bud3p-N (1–673), Bud3p-M (674–1220), Bud3p-C (1221–1636), or Bud3p-MC (674–1220) could (Table 1). Thus, the functional region critical for directing axial budding is located in Bud3p-NM.

**Figure 7:**
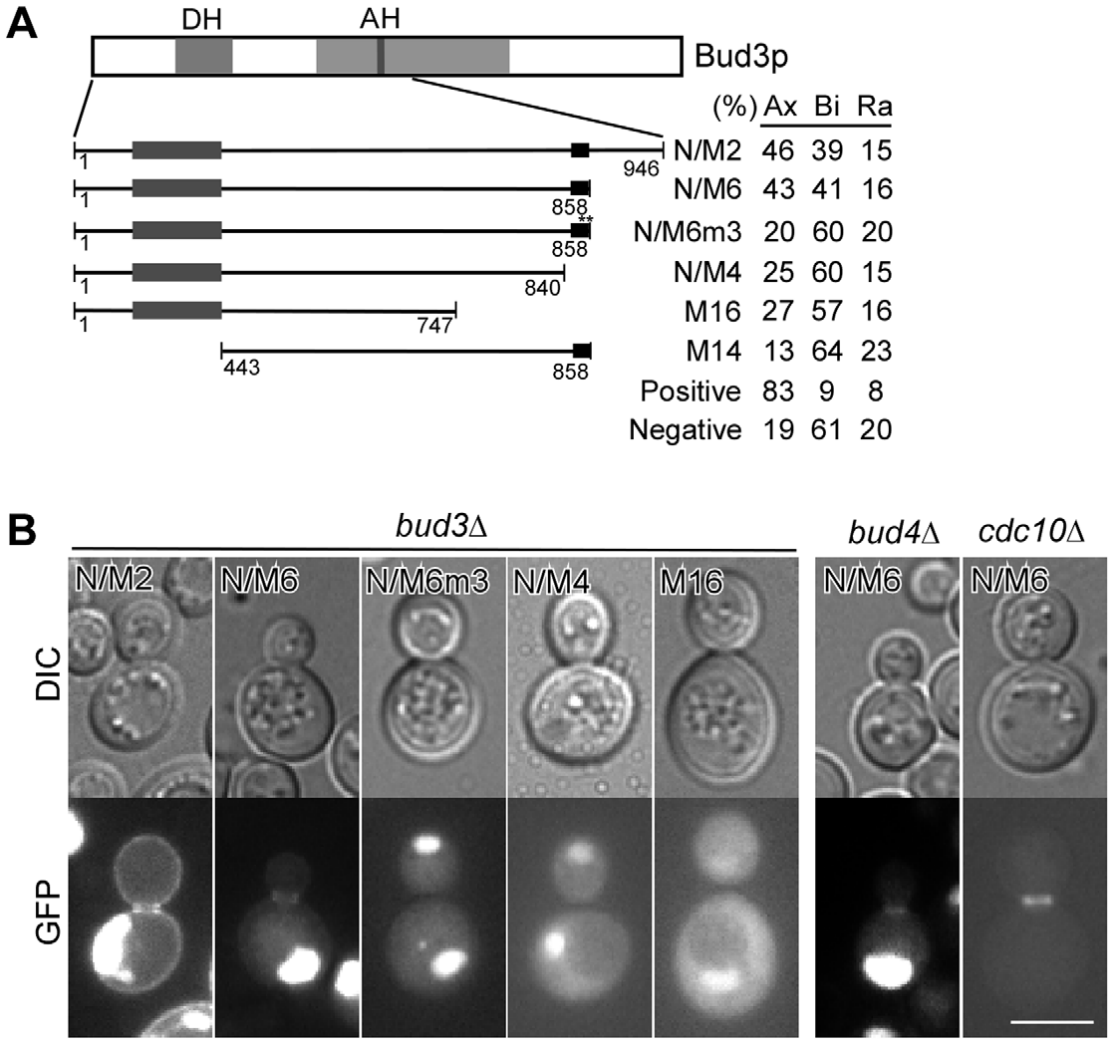
The functional region of Bud3p responsible for axial budding locates in the N-terminal region. (**A**) Restoration of axial budding to *bud3*Δ cells by different Bud3p N-terminal fragments. Cells of strain YEF3570 (a *bud3*Δ) carrying plasmid pUG36-BUD3 fragments were stained for bud scar with Calcofluor. The percentage of cells that bud in axial (Ax), bipolar (Bi), or random (Ra) pattern was scored. The Bud3p-N/M6m3 mutant carries V857A and F858A mutations (shown by two asterisks) in the amphipathic helix. Positive control, pUG36-BUD3; negatvive control, pUG36 empty vector. At least 200 cells that exhibit three or more bud scars were counted. (**B)** Left panel, subcellular localization of representative Bud3p N-terminal fragments shown as in (**A**). Bud3p-N/M6m3 carries V857A and F858A mutations. Right panel, localization of Bud3p-N/M6 in strains YEF3572 (a *bud4*Δ) and YEF4601 (a *cdc10*Δ). Bar, 5 µm.

We then truncated Bud3p-NM to narrow down the functional region. Bud3p-N/M2 (1–946) and Bud3p-N/M6 (1–858), two fragments that carry the region 1–858, could partially restore axial budding to *bud3*Δ cells (Fig. 7A). In contrast, shorter fragments such as Bud3p-N/M4 (1–840), Bud3p-M16 (1–747), and Bud3p-M14 (443–858) could not restore axial budding. Thus, the functional region of Bud3p in axial budding locates in the N-terminal portion of the protein, 1–858.

As expected, Bud3p-N/M2 and Bud3p-N/M6 that could partially restore axial budding localized to the bud neck, whereas Bud3p-N/M4 and Bud3p-M16 that could not restore axial budding did not target to the bud neck (Fig. 7B, left panel). However, the efficiency of bud neck targeting by Bud3p-N/M2 and Bud3p-N/M6 appears to be decreased compared to full-length Bud3p, which may explain the reduction in the percentage of cells that bud axially. Bud3p-N/M6 could also localize to the bud neck in *bud4*Δ cells and *cdc10*Δ cells (Fig. 7B, right panel), suggesting that the bud neck localization of the N-terminal portion (1–858) of Bud3p is mediated by a bud neck-localized protein other than Bud4p and Cdc10p.

In addition to the bud neck, Bud3p-N/M2 and less prominently, Bud3p-N/M6, also displayed a PM localization (Fig. 7B, left panel). The PM localization is mediated by the amphipathic helix 850–858 since the Bud3p-N/M6m3 mutant that carries mutations in two hydrophobic residues (V857A, F858A) in the amphipathic helix did not display PM localization (Fig. 7B, left panel). Surprisingly, the same mutations in Bud3p-N/M6 also eliminated its bud neck localization (Fig. 7B, left panel, N/M6m3). Moreover, the Bud3p-N/M6m3 mutant failed to restore axial budding to *bud3*Δ cells (Fig. 7A), presumably due to a failure in bud neck targeting. Thus, the amphipathic helix (850–858) appears to play an important role in axial budding by promoting Bud3p targeting to the bud neck.

Within the N-terminal portion of Bud3p, the region 259–442 shares sequence homology to the Dbl-homology (DH) domain found in many Rho GEFs [22]. DH domains in Bud3p orthologs from *N. crassa* and *A. nidulans* have been shown to activate Rho4 GTPase and play an essential role in cytokinesis [20], [21]. To investigate whether Bud3p's DH domain is implicated in axial budding, the DH domain (259–442) was deleted in full-length Bud3p. We found that Bud3p-FLΔDH mutant still could restore axial budding to *bud3*Δ cells (Table 1). Thus, the DH domain in Bud3p appears to be dispensable for axial budding.

**Figure 8:**
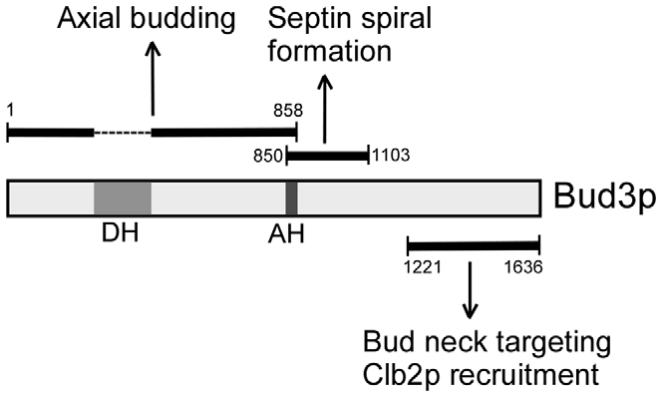
Bud3p controls axial budding and interacts with the septins via different regions. DH, Dbl-homology domain; AH, amphipathic helix.

Taken together, our results indicate that Bud3p's role in axial budding depends on the N-terminal region 1–858, which slightly overlaps with the middle portion 850–1103 involved in septin spiral formation by 9 amino acids, *i.e.* the amphipathic helix 850–858 (Fig. 8). The DH domain within 1–858 is dispensable for axial budding.

## Discussion

### Role of excess Bud3p in the formation of ectopic septin structures

In a number of yeast mutants with defects in septin organization, ectopic septin structures, such as patches, are often observed at the tips or on the periphery of elongated buds [28], [29], [30]. In Bud3p-overexpressing cells, besides septin patches, elaborate spiral-like septin structures are prominent in the elongated buds [16]. In this study, we reported a new type of ectopic septin structures – small rings of ∼1 µm in diameter and short arcs in cells overexpressing Bud3p. Interestingly, septin spirals and small rings both contain Bud3p, suggesting that spirals and rings are both composed of well assembled septin filaments.

Similar septin structures in the forms of small rings and short arcs have been observed previously in yeast cells overexpressing Bni4p, the yeast *Ashbya gossypii*, and cultured fibroblasts during ruffling or treated with an F-actin depolymerizing drug [31], [32], [33], [34]. The diameters of observed septin rings are ∼1 µm, 1 µm, and 0.7 µm, respectively, comparable to the rings of ∼1 µm in Bud3p-overexpressing cells. The spiral-like septin structures, however, are observed in the elongated buds of yeast cells overproducing the *Candida albicans* protein Int1p [35]. Except for *Ashbya gossypii* and ruffling fibroblasts, these ectopic septin structures are all detected under non-physiological conditions. In each case, the septin collars at the bud neck of yeast cells or the long septin bundles in fibroblasts are disrupted as a result of excess Bud3p, Bni4p, or Int1p proteins or the disassembly of actin stress fibers. Most notably, the perpetrators (Bud3p, Bni4p, Int1p, and actin stress fibers) of septin disorganization all appear to bind or associate with septin filaments. Bni4p is a bud neck localized scaffold protein that interacts with the septin Cdc10p [36]. Int1p, however, interacts with the yeast septins Cdc11p and Cdc12p [35]. Int1p also colocalizes with septin filaments at the bud neck and in ectopic spirals. In this study, we showed that Bud3p interacts with the septins Cdc10p and Cdc11p and colocalizes with septin filaments in ectopic spirals and small rings.

Several lines of evidence suggest that Bud3p's function in this process is independent of its role in axial bud-site selection. First, the functional region of Bud3p for the formation of these structures locates in the region 850–1103, which does not overlap significantly with the region 1–858 responsible for axial bud-site selection. Moreover, overexpression of the overlapping region 850–858 (Bud3p-M9) or the N-terminal portion 1–858 (Bud3p-N/M6) containing 850–858 did not cause septin spiral formation. Second, septin spiral formation does not depend on the presence of other axial landmark components as Bud3p overexpression still caused bud elongation in *axl1*Δ, *axl2*Δ, and *bud4*Δ cells (data not shown). Third, overexpression of either Axl1p or Bud4p did not cause bud elongation [35, our unpublished results]. As for Axl2p, the only transmembrane component in the axial landmark, although we previously observed that overexpression of the cytoplasmic tail of Axl2p, caused mild bud elongation in about 20% of cells, however, no spiral-like septin structures were observed [15]. Therefore, our data suggests that Bud3p's function in septin spiral formation is not shared with other axial landmark components.

The detailed mechanism of how Bud3p overexpression leads to the formation of ectopic septin spirals is not clear. We hypothesize that high-levels of Bud3p may titrate an important bud neck component away, leading to disassociation of septin filaments from the bud neck. As septin filaments purified from mammalian cells are known to have a tendency to bundle and circularize into small rings or spirals of ∼0.7 µm in diameter *in vitro* [31], septin filaments disassociated from the bud neck may slowly assemble into higher-order structures elsewhere in the form of short bars, arcs, rings or spirals. One candidate for this bud neck component is Cdc10p as it interacts with both Bud3p and Bni4p. Cdc10p is known to play a key role in organizing septin filaments at the bud neck by bundling septin polymers into paired filaments [27]. We found that Cdc10p is necessary for septin spiral formation as deletion of *CDC10* completely eliminated septin spirals in Bud3p-overexpressing cells (Fig. 6B). However, it seems that Cdc10p is not the only target of Bud3p action because Bud3p overexpression in *cdc10*Δ cells still caused bud elongation and defective septin organization.

### Multiple regions of Bud3p are implicated in bud neck targeting

Our structure-function study revealed that three regions of Bud3p: the N-terminal portion 1–858, the middle portion 841–1220, and the C-terminal portion 1221–1636, all could localize to the bud neck, suggesting that they may all interact with the septins, either directly or indirectly. But several differences in their behavior are also obvious. First, overexpression of the middle portion or the C-terminal portion causes bud elongation and defective septin organization, but overexpression of the N-terminal portion does not. Second, ectopic septin spirals are present in cells overexpressing the middle portion but not in cells overexpressing the C-terminal portion. The latter displays septin patches at the tip or on the side of elongated buds. Third, the N-terminal portion and the middle portion also localize to the PM whereas the C-terminal portion does not. Thus, the three regions of Bud3p may interact with the septins in different manners, which may provide a fine-tuning mechanism to regulate the proper interaction between Bud3p and the septins at different cell cycle stages.

The N-terminal portion 1–858 of Bud3p could partially restore axial budding to *bud3*Δ cells. The bud neck localization of this region is consistent with its role as an axial landmark in bud-site selection. As for the C-terminal portion 1221–1636, its bud neck localization appears to play a role in cytokinesis by recruiting the B-type cyclin Clb2p, an important cell cycle regulator, to the bud neck [18]. In contrast to the N-terminal and the C-terminal portions, the physiological role of the middle portion 841–1220 is not clear. With its unique capability to cause the formation of septin spirals and rings and also colocalize with them, we speculate that it may normally play a role in septin organization.

### Role of the amphipathic helix of Bud3p in PM localization, septin spiral formation, and axial budding

In this study, we report a novel PM localization of Bud3p. Although the physiological role of the PM localization remains elusive, our study with Bud3p truncation fragments revealed that this localization is mediated by an amphipathic helix (850–858) and two flanking BR motifs. The amphipathic helix and flanking BR motifs are evolutionarily conserved among Bud3p orthologs in different yeast species. It will be interesting to investigate if they also serve as PM targeting motifs. In recent years, amphipathic helices with adjacent BR motifs have been identified as plasma membrane-targeting motifs in a number of proteins including the mammalian proteins ARF-1 and RGS4 [23], [25], the bacterial protein MinD [24], [37], the fission yeast protein mid1p [38], and the budding yeast proteins Ste5p, Ste20p, Gic1p, and Gic2p [26], [39], [40]. Thus, it appears to be a common theme that proteins in different species ranging from bacteria to mammals utilize the amphipathic helices and BR motifs for plasma membrane anchoring.

Surprisingly, we found that the amphipathic helix also plays important roles in septin spiral formation and axial budding, since mutations of key hydrophobic residues in the amphipathic helix in Bud3p-M19 (841–1220) and Bud3p-N/M6 abolished their ability to form septin spiral formation and to restore axial budding, respectively (Fig. 5D, Fig.7A). The role of the amphipathic helix in these two processes appears to be mediating the bud neck targeting of these Bud3p fragments (Fig. 5C and Fig. 7B). Thus, it is likely that the amphipathic helix may also be implicated in the interaction with the septins.

### The functional region of Bud3p in axial budding

Bud3p plays a key role in axial budding by acting together with Bud4p to recruit the other two components of the axial landmark, Axl1p and Axl2p, to the bud neck [14], [15]. In this study, we identified the N-terminal portion 1–858 of Bud3p, about half of the entire length, as the functional region in axial budding. This region shares the amphipathic helix 850–858 with the middle portion of Bud3p, 850–1103, which is involved in septin spiral formation. The DH domain (259–442) within Bud3p (1–858) is dispensable for axial budding.

Bud3p's role as a component of the axial landmark relies on its localization to the bud neck. Consistently, we observed that the functional region, 1–858 (Bud3p-N/M6), localizes to the bud neck, in addition to a PM localization. The bud neck localization of Bud3p-N/M6 does not completely depend on Cdc10p or Bud4p, suggesting that other bud neck-localized proteins might be involved.

In contrast to amphipathic helix mutation in Bud3p (1–858), which abolished its ability to restore axial budding to *bud3*Δ cells, the same mutations or deletion of the amphipathic helix in full-length Bud3p did not affect its ability to restore axial budding (data not shown). The discrepancy can be explained by the difference in their localization. The Bud3p (1–858) mutant does not localize to the bud neck (Fig. 7B). However, full-length Bud3p mutants in the amphipathic helix remain localized to the bud neck (Fig. 5B), presumably due to the presence of the C-terminal portion 1221–1636. The data imply that Bud3p (1–858) contains at least two domains, one required for bud neck targeting (interacts with the septins) and the other interacts with other axial landmark components. Future studies will be needed to dissect the specific domains within Bud3p (1–858) that interact with the septins and other components of the axial landmark.

## Materials and Methods

### Genetic methods and strains

Standard culture media and genetic techniques were used except where noted [41]. Yeast strains used in this study are listed in Table S1. *Escherichia coli* strains DH12S (Life Technologies, Gaithersburg, MD) and DH5α (TaKaRa, Japan) were used as hosts for plasmid manipulation. Oligonucleotide primers for PCR were ordered from Sangon Biotech (Shanghai, China). The nucleotide sequences of primers are available upon request.

### Construction of plasmids and yeast strains

To generate plasmids pUG36-BUD3 or BUD3 fragments for protein localization studies, full-length *BUD3* or various *BUD3* fragments was amplified by PCR using plasmid p35-1 or pJC16 (YCp50-pGAL1-BUD3) [6], [16] as template and inserted into *Bam*HI- and *Hin*dIII-digested pUG36 (*CEN*, *URA3*, pMET25-yEGFP3).

Plasmid pEGKT316 that was used to overexpress *BUD3* or *BUD3* fragments was generated by inserting the ∼2 kb *Nco*I-*Bam*HI fragment of plasmid pEGKT carrying 3′-half of *URA3* gene, *GAL1*UAS, *CYC1* promoter, and *GST* into pRS316 vector also digested with *Nco*I and *Bam*HI. *BUD3* or *BUD3* fragments from pUG36-based plasmids were cut with *Bam*HI and *Hin*dIII and ligated into pEGKT316 to generate pEGKT316-BUD3 or BUD3 fragments.

BUD3 mutants in the amphipathic helix (850–858) – BUD3-FLm2 (F850-851A,V854A,L855A), BUD3-FLm3 (V857A, F858A) as well as BUD3-FLΔDH mutant with a deletion in the DH domain (259–442) were generated by overlapping PCR. Two BUD3-M19 mutants–BUD3-M19m2 (F850-851A,V854A,L855A) and BUD3-M19m3 (V857A, F858A) were amplified by PCR from BUD3-FLm2 and BUD3-FLm3 mutants, respectively. The BUD3-M16-ΔDH mutant was amplified by PCR from BUD3-FLΔDH.

For examining the localization of the septin Cdc3p, chromosomal *CDC3* gene in strain YEF473A was tagged with GFP or mCherry by integrating *Bgl*II-linearlized plasmid YIp128-CDC3-GFP or YIp128-CDC3-mCherry [15] at *CDC3* locus to generate strains JGY881 and JGY1783, respectively. Complete deletion of *BUD3*, *CDC10* and *SHS1* genes was constructed in YEF473A by using the PCR-based method with pFA6a-His3MX6 or pFA6a-KanMX6 template [42]. For GST pull-down assay, septin genes *CDC10*, *CDC11*, *CDC12*, and *SHS1* in strain YEF3570 (a *bud3*Δ) were tagged with GFP at their C-terminus by using the PCR-based method with pFA6a-GFP(S65T)-TRP1 template [42] to generate strains JGY2019, JGY2081, JGY2018, and JGY2020, respectively. *CDC3* was tagged with GFP in strain YEF3570 (a *bud3*Δ) using the integrative plasmid YIp128-CDC3-GFP to generate strain JGY2021.

### Microscopy

An Olympus BX51 microscope (Tokyo, Japan) and a Retiga 2000R CCD camera (QImaging Corporation, Canada) were used to visualize cell morphology and GFP-tagged proteins by differential interference contrast (DIC) and fluorescent microscopy. The images were acquired using QCapture Suite (QImaging Corporation, Canada). Image processing was performed with ImagePro Plus (Glen Mills, PA). The budding pattern of a yeast strain was determined by staining the bud scars with calcofluor white (Sigma-Aldrich, F3543). At least 200 cells that exhibit three or more bud scars were counted. For staining of yeast cells for DNA, cells were incubated with 1.0 µg/ml of Hoechst 33258 (Polysciences, Inc. Warrington, PA) for 20 min at 24°C and washed twice with 1×PBS buffer.

### GST pull-down assay

The assay follows a previously described protocol [15]. Yeast strains carrying pEGKT316 vector or pEGKT316-BUD3 were grown at 30°C to A600 of 0.3∼0.4 in 100 ml of SC-Ura medium containing 2% raffinose. Galactose was added to a final concentration of 2% and the cultures were grown for another 4 h to induce the expression of GST and Bud3p-GST fusion proteins. GST-tagged proteins were pulled down with glutathione-Sepharose beads from equal amounts of cell lysates. Primary antibodies used were mouse monoclonal antibodies against GST and GFP (Covance Research Products, Richmond, CA). Secondary antibody was horseradish peroxidase-conjugated goat anti-mouse IgG. Standard immunoblotting procedure was used.

## Supporting Information

Table S1 Yeast strains used in this study.
(DOC)

Figure S1 Expression of Bud3p-GFP fusion proteins in yeast cells. Cells of strain YEF3570 (a *bud3*Δ) carrying plasmid pUG36-BUD3 or pUG36-BUD3 fragments were grown in SC-Ura medium. Cell lysates prepared from the yeast strains were separated by 7.5% SDS-PAGE and immunoblotted with anti-GFP antibodies. FL, Bud3p full-length (1–1636 a.a.), Bud3p-NM (1–1220), Bud3p-MC (674–1636), Bud3p-N (1-673), Bud3p-M (674–1220), Bud3p-C (1221–1636), GFP (pUG36 vector).
(TIF)
