# Supplementary material for: Identification of an Amphipathic Helix Important for the Formation of Ectopic Septin Spirals and Axial Budding in Yeast Axial Landmark Protein Bud3p
Source: PLoS One. 2011 Mar 8;6(3):e16744. doi: 10.1371/journal.pone.0016744 (PMC3050797; doi:10.1371/journal.pone.0016744)
Supplement: Table S1 — Yeast strains used in this study. (DOC) [file pone.0016744.s001.doc]

**Table S1.** Yeast strains used in this study.

| Name | Genotype | Source |
| --- | --- | --- |
| YEF473A | a *his3 leu2 lys2 trp1 ura3* | [43] |
| YEF3570 | a *his3 leu2 lys2 trp1 ura3 bud3*Δ*::HIS3* | J. Pringle |
| YEF3572 | a *his3 leu2 lys2 trp1 ura3 bud4*Δ*::HIS3* | J. Pringle |
| YEF4601 | a *his3 leu2 lys2 trp1 ura3 cdc10*Δ*::KanMX6* | E. Bi |
| JGY881 | a *his3 leu2 lys2 trp1 ura3 CDC3-GFP:LEU2* | See text |
| JGY1783 | a *his3 leu2 lys2 trp1 ura3 CDC3-mCherry:LEU2* | See text |
| JGY2018 | a *his3 leu2 lys2 trp1 ura3 bud3*Δ*::HIS3 CDC12-GFP:TRP1* | See text |
| JGY2019 | a *his3 leu2 lys2 trp1 ura3 bud3*Δ*::HIS3 CDC10-GFP:TRP1* | See text |
| JGY2020 | a *his3 leu2 lys2 trp1 ura3 bud3*Δ*::HIS3 SHS1-GFP:TRP1* | See text |
| JGY2021 | a *his3 leu2 lys2 trp1 ura3 bud3*Δ*::HIS3 CDC3-GFP:LEU2* | See text |
| JGY2081 | a *his3 leu2 lys2 trp1 ura3 bud3*Δ*::HIS3 CDC11-GFP:TRP1* | See text |
