# Supplementary figures and images for: Identification of an Amphipathic Helix Important for the Formation of Ectopic Septin Spirals and Axial Budding in Yeast Axial Landmark Protein Bud3p
Source: PLoS One. 2011 Mar 8;6(3):e16744. doi: 10.1371/journal.pone.0016744 (PMC3050797; doi:10.1371/journal.pone.0016744)

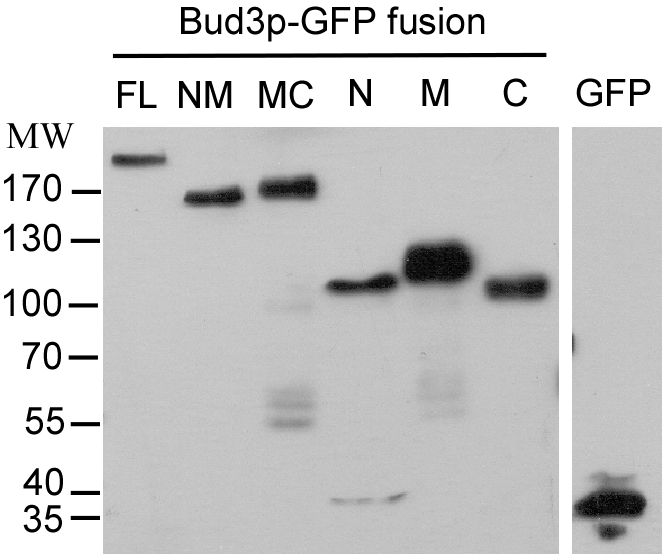

Supplement: Figure S1 — Expression of Bud3p-GFP fusion proteins in yeast cells. Cells of strain YEF3570 (a bud3Δ) carrying plasmid pUG36-BUD3 or pUG36-BUD3 fragments were grown in SC-Ura medium. Cell lysates prepared from the yeast strains were separated by 7.5% SDS-PAGE and immunoblotted with anti-GFP antibodies. FL, Bud3p full-length (1–1636 a.a.), Bud3p-NM (1–1220), Bud3p-MC (674–1636), Bud3p-N (1-673), Bud3p-M (674–1220), Bud3p-C (1221–1636), GFP (pUG36 vector). (TIF) [file pone.0016744.s002.tif]
